# Supplementary material for: Control of Spin-Wave Propagation using Magnetisation Gradients
Source: Sci Rep. 2018 Jul 23;8:11099. doi: 10.1038/s41598-018-29191-2 (PMC6056527; doi:10.1038/s41598-018-29191-2)
Supplement: Supplementary file 1 — Supplementary Material [file 41598_2018_29191_MOESM1_ESM.pdf]

Control of Spin-Wave Propagation using Magnetisation Gradients

– Supplementary Materials –

Marc Vogel[1,*], Rick Aßmann[1], Andrii V. Chumak[1], Philipp Pirro[1], Burkard Hillebrands[1] & Georg von Freymann[1,2]

[1] Department of Physics and State Research Center OPTIMAS, University of Kaiserslautern, Erwin-Schroedinger-Str. 56, 67663 Kaiserslautern, Germany

[2] Fraunhofer-Institute for Physical Measurement Techniques IPM, Fraunhofer-Platz 1, 67663 Kaiserslautern, Germany

[*] Correspondence and requests for materials should be addressed to M. Vogel (email: mvogel@physik.uni-kl.de)

Refraction in a gradient of the saturation magnetisation.

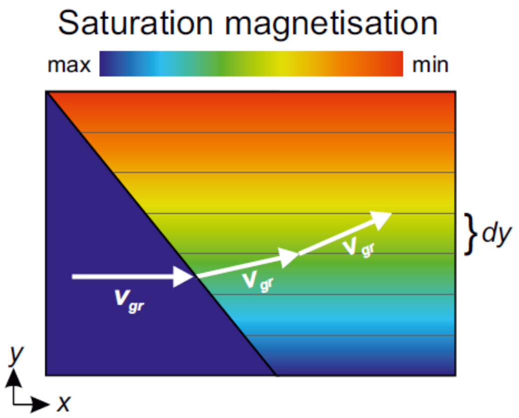

Figure S1 | A simple model illustrates the mode conversion process. The saturation magnetisation gradient can be modelled by infinitely thin slices (thickness: dy) parallel to the x direction. The spin wave is refracted into the y direction at each slice.

The change of $\vec{k}$ in the y direction can be qualitatively understood using the simple model shown in Fig. S1. Next after entering the gradient region, the direction of the group velocity changes depending on the orientation of the interface. As a consequence, the wave propagates into regions with lower saturation magnetisation. The tangential component of $\vec{k}$ – here it is k_x – is conserved at the refraction at an interface of the shown slices. So, only the k_y component can change. How strong this change Δk_y will be is determined via the manifold of possible solutions given by the dispersion relations or isofrequency curves, respectively.

Schematic experimental setup and sample design.

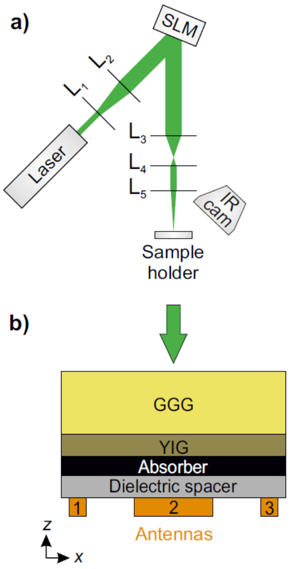

Figure S2 | Scheme of the optical experimental setup **(a)** and the sample consisting of a multilayer system **(b)**.

In contrast to the experimental setup in reference [30] in the main text, no acousto optical modulator was used. A green laser creates temperature or respectively magnetisation gradients (see Fig. S2a): a spatial light modulator changes the local phase fronts of the incoming laser beam to create arbitrary intensity distributions / to reconstruct holograms on the sample which heats up locally. An infrared camera measures the resulting temperature distribution.

The sample used in the experiment is schematically shown in Fig. S1b. It consists of a multilayer system (GGG/YIG/absorber/spacer). The laser light impinges from the GGG (Gadolinium Gallium Garnet) side and is absorbed in YIG (Yttrium Iron Garnet) and the black absorber. The dielectric spacer separates the antennas from the sample in order to minimise the thermal contact.

Additional homogenous light distributions and corresponding local saturation magnetisation. In our experiments, we also investigated homogenous light distributions. Let us discuss three cases to create the magnetisation landscapes (see Fig. S3): a rectangle, a triangle, and a "triangle at the bottom" (meaning that the horizontal edge of the triangle points away from antenna 2). In all the cases presented in Fig. S3 the sample is heated via a uniform intensity distribution. But, even a uniform intensity distribution will create a non-uniform temperature profile – a temperature gradient $\vec{\nabla} T$ and, thus, a magnetisation gradient $\vec{\nabla} M_S(T)$ – due to the intrinsic thermal conductivity of YIG. However, the saturation magnetisation $M_{S,2}(T)$ at antenna 2 is kept constant via adjusting the hologram laser power P_{holo} appropriately.

This is done to compare the cases described in Fig. S3. Antenna 1 and antenna 3 are kept far away from the heated area, thus $M_{S,1}$ and $M_{S,3}$ are almost equal and correspond approximately to the value at room temperature.

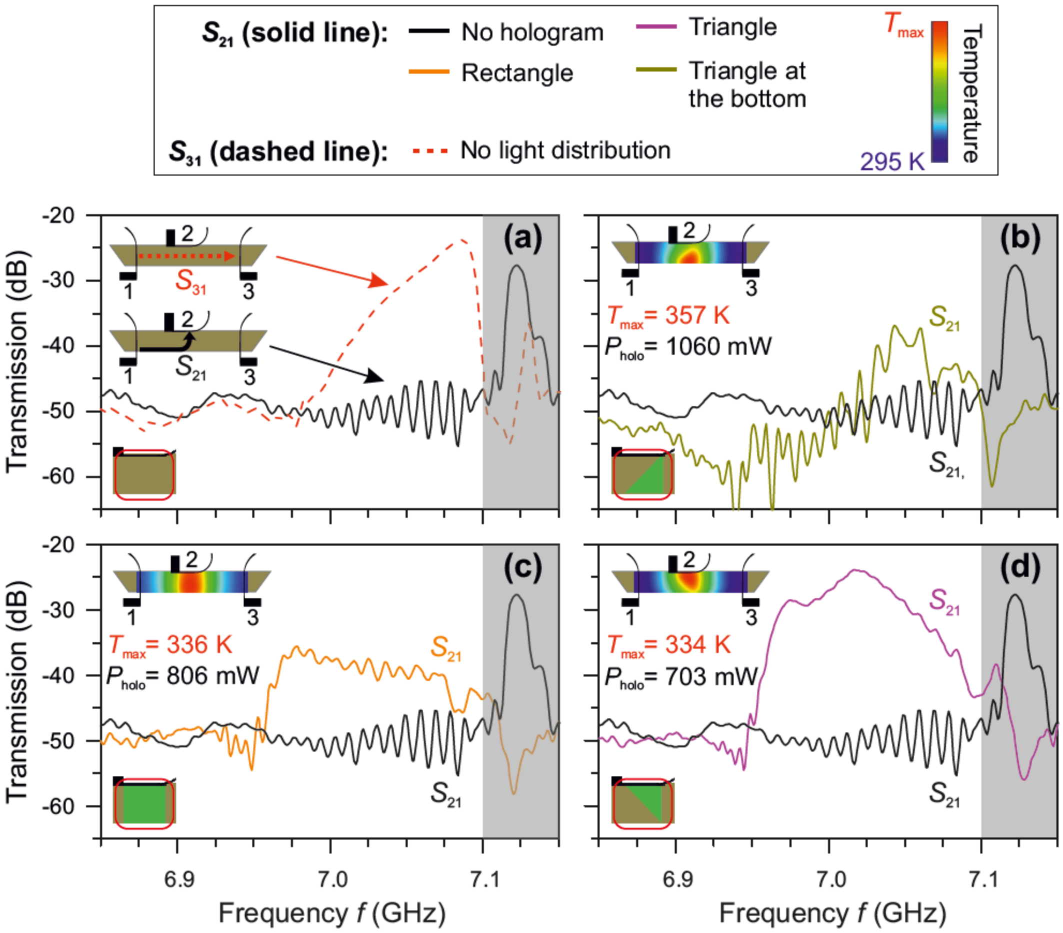

Figure S3 | The transmission characteristics for different light intensity distributions (**a**: no illumination, **b**: triangle at the bottom, **c**: rectangle, **d**: triangle) are shown versus the spin-wave frequency. The light patterns are applied in the centre between antenna 1 and 3 – at the position of antenna 2 ("illumination area" in Fig. 1c) – and the corresponding spin-wave transmission (S_{21} and S_{31}) is measured. The S_{21} (S_{31}) parameter is shown as straight (dotted) line and depicts the transmission of spin waves form antenna 1 to 2 (1 to 3). The light-induced temperature distributions are depicted as colour code (insets, blue: cold, red: hot). $T_{\max}$ is the maximal temperature of the colour scale and corresponds to the individual laser power P_{holo}. The grey area (7.10 – 7.15 GHz) is above the ferromagnetic resonance frequency for an unheated sample (see the S_{31} parameter) and is not of interest in this work.

For the "triangle at the bottom", one clearly observes a weak S_{21} transmission in the frequency range from 7.025 to 7.075 GHz. However, magnetostatic spin waves are strongly anisotropic and a change in the propagation direction should occur simultaneously with a change in the type of spin-wave mode. If we rotate the triangle

counter clockwise by 90 degrees (triangle case) the detected signal at antenna 2 increases drastically. Even the rectangle shows a weak mode conversion. In the latter case, no spin-wave mode conversion is expected since the symmetry of the system is not broken by the laser intensity distribution in the y direction. But regarding the temperature distribution (Fig. S4), the temperature drops down because of von Neumann boundary conditions at the lower edge of the waveguide (at y=0.0 mm). Thus, the symmetry in the saturation magnetisation is broken nonetheless. The spin-wave conversion efficiency can be increased further by directly shaping the gradient – as shown in the main text. This is easily realised in our experimental setup by modifying the hologram [30].

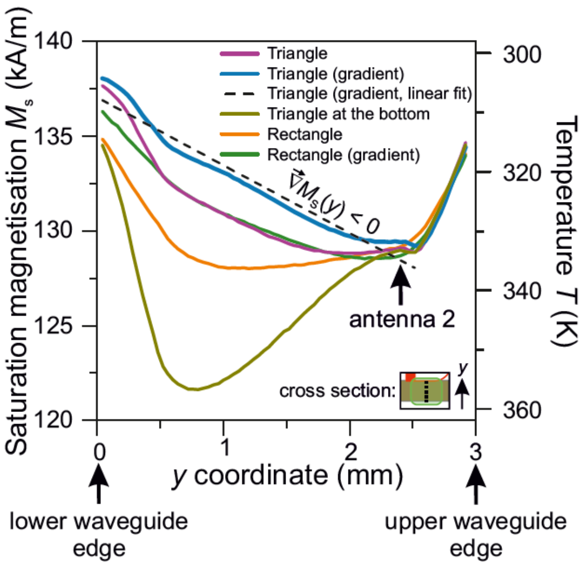

Figure S4 | The profiles of the saturation magnetisation along the y coordinate (over the waveguide width) at the centre of the illumination area (see inset, black dotted line) are shown for the different light distributions and laser powers used in Fig. S3. Gradients in the saturation magnetisation with a negative slope cause the mode conversion. In Fig. 2c only the cases with an intensity gradient are presented.

Micromagnetic simulations.

1) **Dispersion relations for the saturation magnetisation gradient used in the numeric calculations.** The M_S variation/distribution reaches from about 123 kA/m (maximal heating in the experiment) to 141 kA/m (at room temperature). Thus, the mode conversion area is enlarged compared to Fig. 1b. Mode conversion is possible for an excitation frequency of 7.0 GHz, which is chosen exemplarily in the micromagnetic simulations in Fig. 4. The green arrow depicts the wavevector’s transformation inside the gradient area accordingly to the isofrequency curves in Fig. 4a.

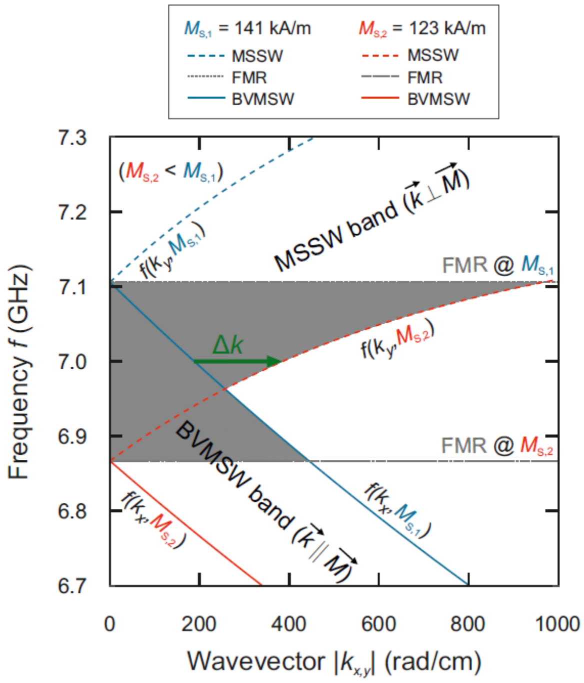

Figure S5 | The dispersion relations of BVMSWs and MSSWs are shown for different values of the saturation magnetisation corresponding to the micromagnetic simulations shown in Fig. 4. The change in the wavevector's magnitude due to the conversion process is depicted as green arrow.

2) Micromagnetic simulations for pure BVMSW behaviour. If the spin waves are excited at a frequency below the ferromagnetic resonance frequency for the lowest saturation magnetisation, pure BVMSW behaviour is expected. Exemplarily, for f=6.8 GHz MSSWs cannot be excited for the minimal saturation magnetisation of around 123 kA/m since the decrease in M_S is too small (see the dispersion relations in Fig. S5). Consequently, no isofrequency curves for MSSWs are occurring above the line corresponding to the critical angle φ_c. The spin-wave propagation in the gradient area is the same as in Fig. 4 for waves entering the $\vec{\nabla} M_S$ region below $M_{S,FMR}$. In the case of a rectangular magnetisation gradient area (Fig. S6a), the spin waves propagating at $M_S \approx 141\,\mathrm{kA/m}$ enter the gradient at a certain value $M_{S,in}$ defined by the coordinate in the direction perpendicular to the propagation direction. Thus, the spin-wave wavevector, and, respectively, the wavelength change differently in different positions over the waveguide's width resulting in a bending of the phase fronts and in an adjustment of the group velocity's direction. The corresponding isofrequency curves illustrate how this process is happening: the spin waves at $M_S \approx 141\,\mathrm{kA/m}$ propagate into the gradient region at $M_{S,in}$. The tangential component of $\vec{k}$ (with respect to the interface) is conserved and only k_x is changed. After entering $\vec{\nabla} M_S(x,y)$, k_x is conserved and only k_y is modified since

the translational symmetry of the system is broken due to the magnetisation gradient in the y direction. The change Δk_y is due to refraction of the spin waves in the gradient area, which is much larger than the wavelength [29]. As a consequence, $\vec{k}$ rotates in the x-y-plane. In the triangular case (Fig. S6b) additional refraction at the interface to the gradient area occurs, which changes the x and y component of the wavevector.

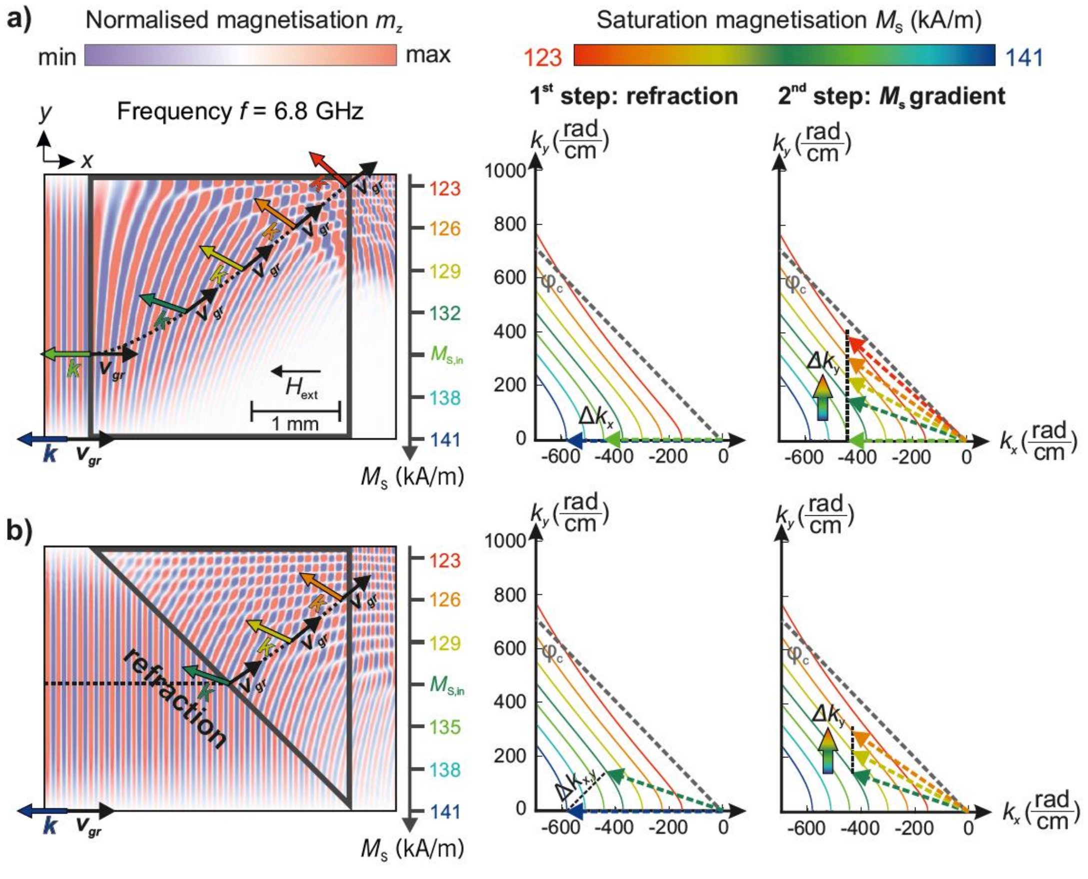

Figure S6 | Reference simulations for f=6.8 GHz (pure BVMSW behaviour) in addition to Fig. 4 in the main text.

3) ***Micromagnetic simulations – no upper waveguide edge / energy flow.*** In this case, the waveguide is enlarged at the upper edge. Outside of the black area (gradient area), the saturation magnetisation M_S is kept constant (~141 kA/m in the left region, ~123 kA/m in the top region). Since no reflections are occurring at the top, the phase fronts can be seen clearer (no superposition of incoming and reflected waves). Furthermore, a fast Fourier transform (FFT) in time shows how the energy flows through the gradient region. On the left side of the gradient region a standing wave is formed. For a frequency f=7.0 GHz, the triangular hologram is much more effective compared to a rectangular one.

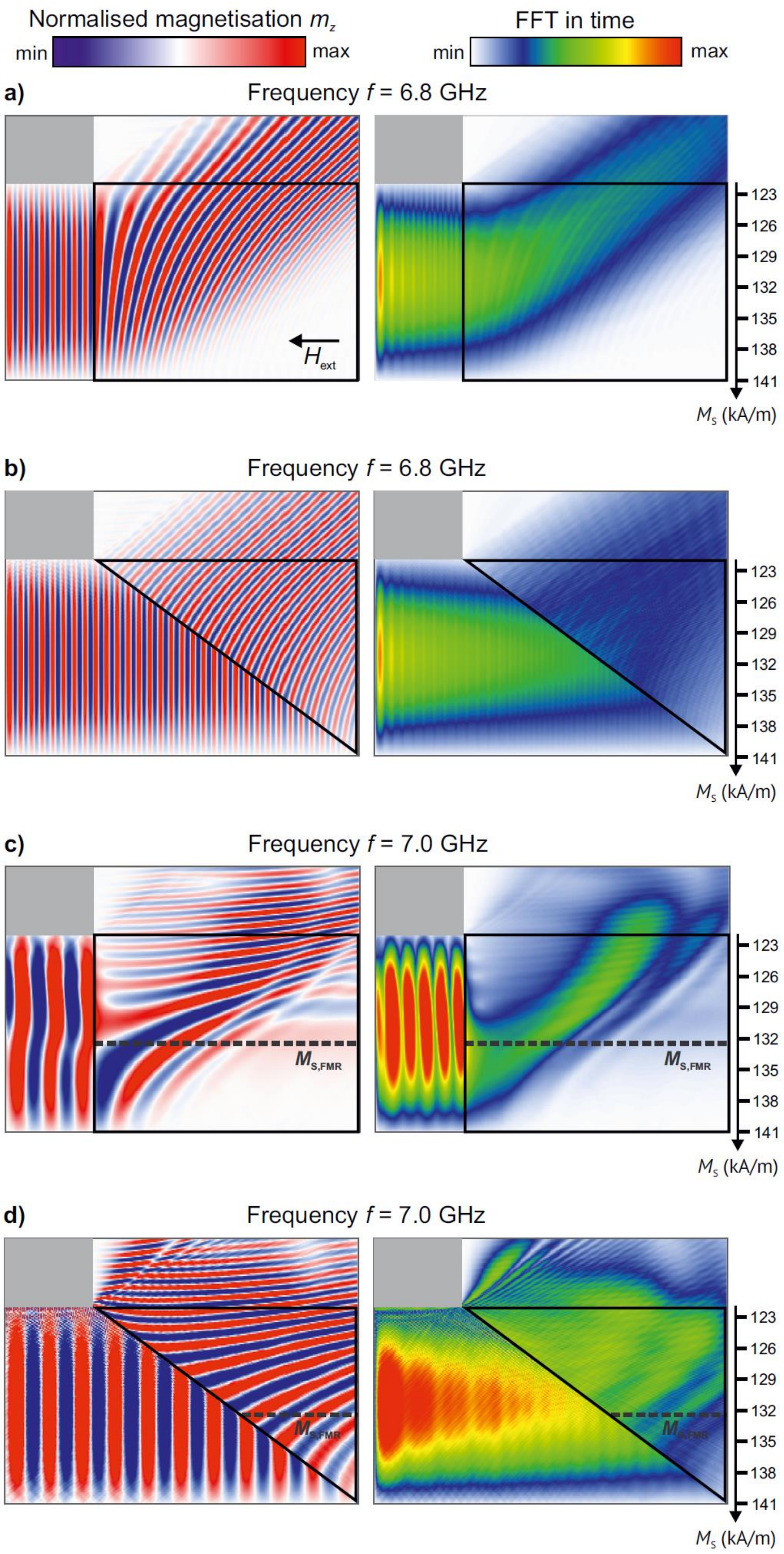

Figure S7 | Micromagnetic simulations (left column) for the frequencies 6.8 GHz (**a**, **b**) and 7.0 GHz (**c**, **d**) and different magnetisation landscapes (rectangle: **a**, **c** & triangle: **b**, **d**). The energy flow is shown as fast Fourier transformation in time in the right column. The M_S gradient is the same as in Fig. 4.

4) ***Micromagnetic simulations – qualitative determination of the conversion efficiency.*** We used the simulations with no upper waveguide edge (see above) to determine the conversion efficiency qualitatively. Therefore, we calculated the mean value of the precessing dynamic magnetisation's *z*-compontent in the extended part of the waveguide. We only use the cells of the simulation area which lie above the critical angle (46°, see below). The simulations for a triangular saturation magnetisation distribution (red curve) fit the data very well for frequencies below 6.9 GHz and above 7.05 GHz. In contrast, the orange curve (rectangular magnetisation landscape) corresponds very well for frequencies in between these two values. Since the experimental temperature distribution is not as sharp as in the simulations, the measured curve of the efficiency is a mixture of both simulations.

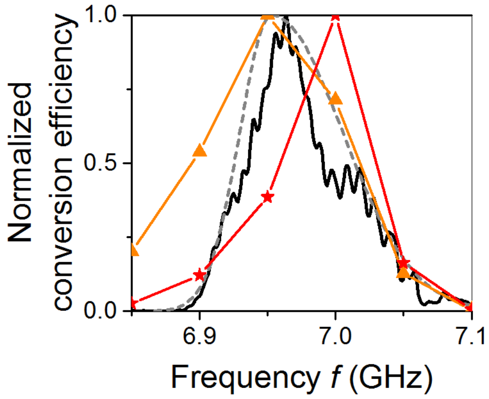

Figure S8 | The experimentally determined efficiency shown in Fig. 4a is compared with the simulations (orange: rectangular magnetisation landscape; red: triangular magnetisation landscape).

The critical angle and its dependency on the saturation magnetisation. The critical angle φ_c defines if MSSW modes can exist for the propagation into a certain direction φ. More information concerning φ_c can be found in e.g. reference [36]. It is defined as:

$$\varphi_c(T) = \arctan\left(\sqrt{\frac{H_{ext}}{M_S(T)}}\right)$$

The saturation magnetisation dependency of the critical angle is shown in the following plot for $H_{ext} = 143\ \mathrm{kA/m}$ (equals $\mu_0 H_{ext} \approx 180\ \mathrm{mT}$):

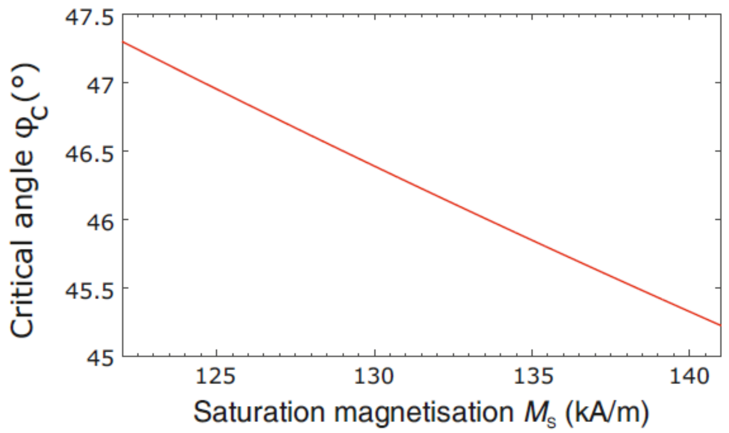

Figure S9 | The critical angle as a function of the saturation magnetisation.

The average value over the shown range of M_S is approximately 46 degrees.

Additional movies. In Fig. 4 and S6, the numerical calculations of the spin-wave propagation in different gradients of the saturation magnetisation is shown 120 ns after the excitation. These additional movies show the full micromagnetic simulations for 0 – 120 ns. The time step between every frame of the movie is 1 ns. The spin-wave is excited in the centre of the waveguide. The landscape of the saturation magnetisation is placed on the right side of the excitation area. On the left side, the uninfluenced spin wave is shown as a reference.
